# Impulsivity across severe mental disorders: a cross-sectional study of immune markers and psychopharmacotherapy

**DOI:** 10.1186/s12888-023-05154-4

**Published:** 2023-09-07

**Authors:** Gabriela Hjell, Jaroslav Rokicki, Attila Szabo, René Holst, Natalia Tesli, Christina Bell, Thomas Fischer-Vieler, Maren Caroline Frogner Werner, Synve Hoffart Lunding, Monica Bettina Elkjær Greenwood Ormerod, Ingrid Torp Johansen, Srdjan Djurovic, Thor Ueland, Ole Andreas Andreassen, Ingrid Melle, Trine Vik Lagerberg, Lynn Mørch-Johnsen, Nils Eiel Steen, Unn Kristin Haukvik

**Affiliations:** 1 NORMENT, Division of Mental Health and Addiction, Oslo University Hospital & Institute of Clinical Medicine, University of Oslo, Oslo, Norway; 2 Department of Psychiatry & Department of Clinical Research, Østfold Hospital, Grålum, Norway; 3 Centre of Research and Education in Forensic Psychiatry, Oslo University Hospital, Oslo, Norway; 4 K.G. Jebsen Center of Neurodevelopmental Disorders, University of Oslo, Oslo, Norway; 5 Department of Biostatistics, Institute of Basic Medical Sciences, University of Oslo, Oslo, Norway; 6 Department of Medical Genetics, Oslo University Hospital, Oslo, Norway; 7 NORMENT, Department of Clinical Science, University of Bergen, Bergen, Norway; 8 Institute of Clinical Medicine, University of Oslo, Oslo, Norway; 9 Research Institute of Internal Medicine, Oslo University Hospital, Oslo, Norway; 10 K.G. Jebsen Thrombosis Research and Expertise Center, University of Tromsø, Tromsø, Norway; 11 Department of Adult Psychiatry, Institute of Clinical Medicine, University of Oslo, Oslo, Norway

**Keywords:** Impulsiveness, Cytokines, Interleukin-1, Tumor necrosis factor, Chemokine CCL5, Psychopharmacology, Lithium, Antidepressants, Schizophrenia, Bipolar disorder

## Abstract

**Background:**

Impulsivity is a transdiagnostic feature linked to severe clinical expression and a potential target for psychopharmacological strategies. Biological underpinnings are largely unknown, but involvement of immune dysregulation has been indicated, and the effects of psychopharmacological agents vary. We investigated if impulsivity was associated with circulating immune marker levels and with a range of psychopharmacological treatment regimens in severe mental disorders.

**Methods:**

Impulsivity was assessed in a sample (*N* = 657) of patients with schizophrenia or schizophreniform disorder (SCZ) (*N* = 116) or bipolar disorder (BD) (*N* = 159) and healthy participants (*N* = 382) using the Barratt Impulsiveness Scale (BIS-11) questionnaire. Plasma levels of systemic immune markers (RANTES, IL-1RA, IL-18, IL-18BP, sTNFR-1) were measured by enzyme immunoassays. Patients underwent thorough clinical assessment, including evaluation of psychotropic medication. Associations were assessed using linear regressions.

**Results:**

Impulsivity  was positively associated with SCZ (*p* < 0.001) and BD (*p* < 0.001) diagnosis and negatively associated with age (*p* < 0.05), but not significantly associated with any of the circulating immune markers independently of diagnostic status. Among patients, impulsivity was negatively associated with lithium treatment (*p* = 0.003) and positively associated with antidepressant treatment (*p* = 0.011) after controlling for diagnosis, psychotropic co-medications, manic symptoms, and depressive symptoms.

**Conclusions:**

We report elevated impulsivity across SCZ and BD but no associations to systemic immune dysregulation based on the current immune marker selection. The present study reveals associations between impulsivity in severe mental disorders and treatment with lithium and antidepressants, with opposite directions. Future studies are warranted to determine the causal directionality of the observed associations with psychopharmacotherapy.

**Supplementary Information:**

The online version contains supplementary material available at 10.1186/s12888-023-05154-4.

## Background

Schizophrenia and bipolar disorder are severe mental disorders with overlapping clinical presentations, environmental risk factors, and polygenic architectures [1, 2]. The development of a severe mental disorder affects quality of life and functioning, although illness course varies substantially between individuals [3]. Impulsivity can be conceptualized as a tendency to react without considering the consequences [4]. Elevated impulsivity has been demonstrated in bipolar and schizophrenia spectrum disorders as well as in a range of other mental disorders such as attention-deficit hyperactivity disorder, borderline and antisocial personality disorders, and intermittent explosive disorder [5–7]. Impulsivity has been linked to severe clinical expression, including suicidality, aggression, and early onset of the disorder [7–9]. Thus, it has been proposed that patients across psychiatric diagnostic categories that express high impulsivity levels may benefit from preventive and therapeutic strategies targeting impulsivity [7, 8]. However, the biological underpinnings of impulsivity are largely unknown, which imposes limitations to the development of optimized treatment and prevention of adverse outcomes.

A growing body of evidence points to involvement of the immune system in mental health and illness. Clinical genome-wide association studies and transcriptome-wide approach in human brain tissue have suggested a role of immune pathways across severe mental disorders [10, 11]. Furthermore, elevations of circulating inflammatory immune markers have been demonstrated in schizophrenia, bipolar, as well as major depressive disorder [12]. Intriguingly, impulsivity and impulsivity-related clinical phenomena such as agitation and aggression have been linked to disturbances in inflammatory pathways, both in the general population [13] and across mental disorders [14–19]. Specifically, the chemokine Regulated on activation normal T cell expressed and secreted (RANTES), interleukin (IL)-1 family, and tumor necrosis factor (TNF) pathways have been proposed as pathophysiological candidates of impulsivity based on studies among individuals with alcohol dependence [20] and suicidal behavior [21], as well as on rodent models [22]. The IL-1 family signaling pathways include immune markers such as IL-1β, IL-1 receptor antagonist (IL-1RA), IL-18, and IL-18 binding protein (IL-18BP), while markers such as TNF and soluble TNF receptor 1 (sTNFR1) belong to the TNF superfamily. The IL-1 family and TNF signaling pathways are involved in the coordination of innate immune responses and have potent pro-inflammatory properties [23, 24]. As ligands in these immune marker superfamilies circulate at levels just above the detection limit of commercially available assays, use of surrogate stable markers such as IL-1RA and sTNFR1 can be employed to reliably reflect the activity within IL-1 and TNF systems [25, 26]. Interestingly, the IL-1 family and TNF signaling pathways have been proposed to interplay with neurotransmission and neuronal excitability [27]. Likewise, the inflammatory chemoattractant RANTES, with previously indicated elevated systemic levels in schizophrenia [28, 29], has been suggested to play a neuromodulatory role [30, 31]. However, potential links between these immune pathways and impulsivity, with their possible impact on psychopathology in severe mental disorders, are yet to be determined.

Impulse control impairments are often seen in the context of illness exacerbations such as psychotic or manic episodes, which can be treated with antipsychotics, anticonvulsants, and lithium [32–34]. Intriguingly, animal models have shown impulsivity-lowering effect of lithium [35, 36], paralleled by anti-inflammatory effects (i.e., decrease of RANTES and IL-1β levels in plasma and brain tissue) [22]. Impulsivity-lowering effects of antipsychotics [37] and anticonvulsants [38] have also been indicated, and a role of dopaminergic and serotoninergic signaling has been suggested [39]. Further, despite that adjunctive psychopharmacotherapy with antidepressants is broadly used in clinical practice across bipolar and schizophrenia spectrum disorders [40, 41], its relationship to impulsivity in severe mental disorders has not been evaluated.

The aim of the present study was to (1) investigate associations between impulsivity and plasma levels of immune markers in a large cross-sectional sample of individuals with and without severe mental disorder and (2) explore links between impulsivity and psychopharmacological treatment in a naturalistic setting. We hypothesized that plasma levels of RANTES, IL-1RA, IL-18, IL-18BP, and sTNFR1 would be positively associated with impulsivity across the diagnostic categories. Further, we hypothesized that antipsychotic, anticonvulsant, and lithium treatment would be negatively associated with impulsivity. Given the sparsity of evidence regarding the relationship between antidepressants and impulsivity in severe mental disorders, the corresponding part of our study was explorative.

## Methods

### Study design and participants

The present study is a cross-sectional investigation of impulsivity in a sample (*N* = 657) of participants recruited between the years 2011 and 2018 through the ongoing Thematically Organized Psychosis (TOP) study at the NORMENT research center, Oslo, Norway. The TOP study enrolls patients with severe mental disorders referred from psychiatric inpatient and outpatient clinics and age- and catchment area matched healthy controls randomly selected from the national population registry. In the patient group (*N* = 275), the main inclusion criterion was a schizophrenia or schizophreniform disorder diagnosis (SCZ, grouped together based on the extensive overlap in clinical features [42] and in accordance with common research practice [43]) or a bipolar disorder diagnosis (BD) assigned according to the Diagnostic and Statistical Manual of Mental Disorders Fourth Edition (DSM-IV) [42]. Further inclusion criteria were age between 18 and 65 years and the ability to give informed consent. The exclusion criteria consisted of pronounced cognitive deficit (IQ scores below 70), history of severe head trauma, neurological disorder, immunological condition, current infection (indicated by medical records, self-report, medication use, or C-reactive protein (CRP) level above 10 mg/L), and use of any immunomodulatory agents. In the healthy participant group (*N* = 382), the presence or history of a severe mental disorder among the participants or their first-degree relatives constituted an additional exclusion criterion.

### Clinical assessment

Participants in the patient group underwent general physical examination, review of medical records, and clinical interviews, including the Structured Clinical Interview for DSM-IV axis I disorders (SCID-I) [44]. The assessments resulted in assigning one of the following diagnoses; SCZ (*N* = 116): schizophrenia (DSM-IV 295.1, 295.3, 295.6, 295.9, *N* = 103), schizophreniform disorder (DSM-IV 295.4, *N* = 13) or BD (*N* = 159): bipolar I (DSM-IV 296.0, 296.4, 296.5, 296.6, 296.7, *N* = 90), bipolar II (DSM-IV 296.89, *N* = 59), bipolar not otherwise specified (DSM-IV 296.80, *N* = 10). Symptom load was evaluated with the Positive and Negative Syndrome Scale (PANSS) [45], the Young Mania Rating Scale (YMRS) [46], and the Calgary Depression Scale for Schizophrenia (CDSS) [47]. The level of functioning was measured according to the Global Assessment of Functioning Split Version (GAF-F) [48]. In addition to a comprehensive review of somatic and psychiatric history, healthy participants were assessed using the Primary Care Evaluation of Mental Disorders [49].

### Impulsivity scores

Impulsivity was measured using the Barratt Impulsiveness Scale (BIS-11) questionnaire [50]. The BIS-11 is commonly used to assess behavioral and personality constructs of impulsivity across general- and patient populations [51]. The BIS-11 consists of 30 items, which are self-evaluated on a 4-point Likert scale. The total score ranges from 30 to 120, with higher scores reflecting higher levels of impulsivity. Internal consistency of the total score has been reported as acceptable [4, 50–53].

### Psychotropic medication

All patients were interviewed about their current pharmacological treatment, and medical records were used to validate the information. The psychopharmacological agents were sorted into the following groups: antipsychotics (olanzapine, risperidone, paliperidone, amisulpride, aripiprazole, clozapine, quetiapine, zuclopenthixol, perphenazine, ziprasidone, chlorprothixene, levomepromazine), anticonvulsants (valproate, lamotrigine, carbamazepine), lithium, and antidepressants (escitalopram, fluoxetine, sertraline, paroxetine, venlafaxine, mirtazapine, mianserin, bupropion). The current dose relative to the defined daily dose (DDD) was calculated for the antipsychotics, anticonvulsants, lithium, and antidepressants, according to the guidelines from the World Health Organization Collaborating Center for Drug Statistics Methodology (https://www.whocc.no/atc_ddd_index/).

### Immune markers

Blood samples were collected using venipuncture and EDTA vials. The median time of the blood sampling was 10 a.m. in the patient group and 3 p.m. among the healthy participants. Plasma was isolated the next working day and stored at -80 °C in the biobank. Samples were not refrigerated during shipment to the biobank. Plasma concentrations of immune markers were measured with enzyme-linked immunosorbent assay (ELISA) methods, using IL-1RA antibodies (Cat#900K474) from PeproTech (Cranbury, NJ, USA) and IL-18 (Cat#DY318-05), IL-18BP (Cat#DY119), sTNFR1 (Cat#DY225), and RANTES antibodies (Cat#DY278) from R&D Systems (Stillwater, MN, USA). RANTES, IL-18 and IL-18BP were analyzed in 2018, while IL-1RA and sTNFR1 were analyzed in a subsample of participants (*N* = 240) in 2013. All analyses were conducted in duplicate in a 384-well format, using a pipetting robot (SELMA, Analytik Jena, Jena, Germany) and a washer dispenser (BioTek, Winooski, VT, USA). Absorption was read by ELISA plate reader (BioTek, Winooski, VT, USA) at 450 nm with 540 nm wavelength correction. The assay sensitivities were: 20 pg/mL for RANTES, 25 pg/mL for IL-1RA, 22 pg/mL for IL-18, 25 pg/mL for IL-18BP, and 20 pg/mL for sTNFR1. In 10 samples (1.5%), levels of RANTES were under the detection limit and were set to 20 pg/mL, while level of IL1-RA was under the detection limit in 1 sample (0.4%) and thus set to 25 pg/mL. Intra- and inter-assay coefficients of variation were below 10% for all analyses. To ensure compliance with the exclusion criteria, samples were screened for serum CRP levels above 10 mg/L, using particle-enhanced immunoturbidimetric methods from Roche Diagnostics (Indianapolis, IN, USA) at the Department of Medical Biochemistry, Oslo University Hospital, Oslo, Norway.

### Statistical analyses

Data were analyzed using the R software package version 4.2.1 (www.R-project.org). Normality of the distributions was assessed by Kolmogorov–Smirnov tests, and differences in Descriptive characteristics were compared across diagnostic categories using Wilcoxon rank-sum tests, Kruskal–Wallis tests with post hoc pair-wise comparisons, or chi-squared tests. Before further analyses, the BIS-11 total scores (measure of impulsivity) were successfully log-transformed to attain normality. Following inspection of the immune marker distributions, extreme values exceeding the first or third quantile by three interquartile ranges or more were removed prior to entry into analyses (1.4% of RANTES, 3.8% of IL-1RA, 0.5% of IL-18, 1.7% of IL-18BP, and 0.4% of sTNFR1). Linear regressions with one immune marker at a time as the independent variable and impulsivity as the dependent variable were employed, while controlling for sex, age, diagnosis (healthy individuals, SCZ, and BD coded as dummy variables), BMI, and smoking status as a dichotomous variable. Further, the relationships between immune markers and impulsivity in the separate diagnostic groups (healthy individuals, SCZ, and BD) were assessed in exploratory analyses, while controlling for sex, age, BMI, and smoking status in the analyses comprising healthy individuals, and controlling for sex, age, BMI, smoking status, psychotropic medication (DDD of antipsychotics, anticonvulsants, lithium, and antidepressants), manic symptoms (YMRS score), and depressive symptoms (CDSS score) in the SCZ analyses and the BD analyses. Next, associations between psychopharmacological treatment and impulsivity were investigated in the patient group. We ran a linear regression with DDD of antipsychotics, anticonvulsants, lithium, and antidepressants as independent variables and impulsivity as the dependent variable, while controlling for sex, age, diagnosis (SCZ versus BD), manic symptoms (YMRS score), and depressive symptoms (CDSS score), and we conducted follow-up analyses in the separate diagnostic groups (SCZ and BD). Standardized residuals, variance inflation factors, and Cook’s distances were inspected to ensure no violation of the model assumptions. All analyses were two-tailed, with a general significance level at 0.05. Based on Bonferroni correction for multiple testing, significance level was set at 0.01 (0.05/5) for the immune marker analyses and at 0.0125 (0.05/4) for the analyses of psychopharmacotherapy.

## Results

### Descriptive characteristics

**Fig. 1:**
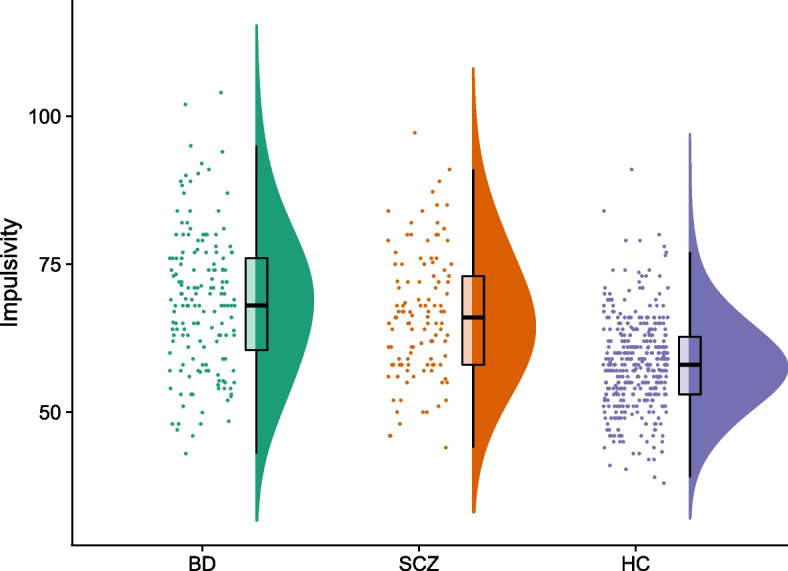
Impulsivity across individuals with severe mental disorders and healthy individuals. BD bipolar disorder; SCZ, schizophrenia or schizophreniform disorder; HC, healthy participant group. Impulsivity displayed as total scores on Barratt Impulsiveness Scale 11

The median of the BIS-11 total score was 66 in the SCZ group, 68 in the BD group, and 58 in the healthy participant group. The BIS-11 total scores were higher in both the SCZ (*p *< 0.001) and the BD (*p* < 0.001) group, compared to the healthy participants, while there were no significant differences in the BIS-11 total scores between the patient groups (*p* = 0.09) (Fig. 1). Patients in the BD group were more often female and had a higher level of functioning, more depressive symptoms, and lower total PANSS scores than patients in the SCZ group. Compared to patients in the SCZ group, patients in the BD group also less often used antipsychotics and more often used anticonvulsants and lithium. Descriptive characteristics are presented in more detail in Table 1, Table 2, and Table S1.

**Table 1:** Demographic and clinical characteristics

	**SCZ**	**BD**	**HC**	**p**^SCZ versus HC^	**p**^BD versus HC^	**p**^SCZ versus BD^
Total *N* = 657	*N* = 116	*N* = 159	*N* = 382			
**N (%)**						
Male	78 (67)	63 (40)	221 (58)	0.07	< 0.001	< 0.001
Antipsychotics	104 (90)	72 (45)	NA	NA	NA	< 0.001
Anticonvulsants	9 (8)	43 (27)	NA	NA	NA	< 0.001
Lithium	5 (4)	27 (17)	NA	NA	NA	0.001
Antidepressants	21 (18)	35 (22)	NA	NA	NA	0.43
**Median (IQR)**						
Age	28 (14)	30 (17)	31 (13)	p ^Kruskal–Wallis test^ = 0.41		
BIS-11 total score	66 (15)	68 (16)	58 (10)	< 0.001	< 0.001	0.09
PANSS total score	62 (18)	42 (11)	NA	NA	NA	< 0.001
CDSS	3 (5)	4 (7)	NA	NA	NA	0.02
YMRS	2 (4)	2 (4)	NA	NA	NA	0.82
GAF-F	42 (16)	60 (19)	NA	NA	NA	< 0.001

*BD* Bipolar disorder, *BIS-11* Barratt Impulsiveness scale, *CDSS* Calgary Depression Scale for Schizophrenia, *GAF-F* Global Assessment of Functioning, *HC* Healthy participant group, *IQR* Interquartile range, *NA* Not applicable, *PANSS* Positive and Negative Syndrome Scale, *SCZ* Schizophrenia or schizophreniform disorder, *YMRS* Young Mania Rating Scale

*p* values based on chi-squared-, Wilcoxon rank-sum-, or Kruskal–Wallis tests with post hoc pair-wise comparisons

**Table 2:** Circulating immune marker levels (pg/mL)

Median (IQR)	**SCZ**	**BD**	**HC**
**RANTES**	76.2 (82.6)	65.6 (60.6)	89.5 (77.3)
**IL-1RA**	297.0 (416.9)	267.5 (359.9)	247.3 (247.0)
**IL-18**	1 358.7 (1 895.2)	1 078.2 (1 369.2)	1 227.1 (1 308.5)
**IL-18BP**	6 285.7 (2 830.6)	5 597.4 (2 136.1)	5 244.2 (2 628.9)
**sTNFR1**	2 174.3 (947.6)	2 398.5 (1 306.4)	1 284.5 (858.0)

### Associations between impulsivity and the immune markers

**Fig. 2:**
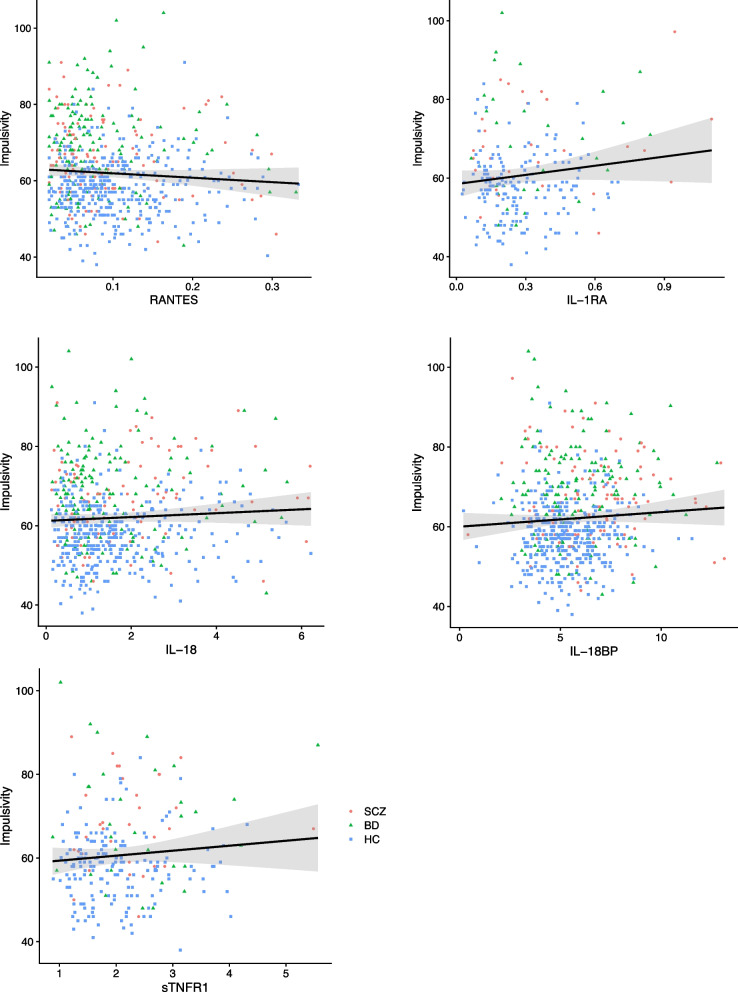
Associations between circulating immune marker levels and impulsivity. X axis: Plasma level of the immune marker (ng/mL). Y axis: Barratt Impulsiveness scale 11, total score. RANTES, Regulated on activation normal T cell expressed and secreted; IL-1RA, Interleukin-1 receptor antagonist; IL-18, Interleukin-18; IL-18BP, Interleukin-18 binding protein; sTNFR1, Soluble tumor necrosis factor receptor 1. SCZ, schizophrenia or schizophreniform disorder; BD bipolar disorder; HC, healthy participant group

In the full model, impulsivity was positively associated with SCZ (*p* < 0.001) and BD (*p* < 0.001) diagnosis and negatively associated with age (*p *< 0.05), while there was no significant association with sex. As shown in Table 3, there were no significant associations between the immune markers and impulsivity in any of the separate diagnostic groups (SCZ, BD, healthy individuals) or in the total sample. Visualization of the relationships between the immune markers and impulsivity is presented in Fig. 2.

**Table 3:** Linear regressions of associations between circulating immune markers and impulsivity

	**Coefficient estimate (99% CI)**	**Standardized coefficient β**	**t**	***p***
**Total sample**^a^				
RANTES	-0.23 (-0.54 to 0.08)	-0.08	-1.93	0.05
IL-1RA	0.03 (-0.15 to 0.21)	0.03	0.39	0.70
IL-18	0.004 (-0.01 to 0.02)	0.03	0.74	0.46
IL-18BP	0.001 (-0.01 to 0.01)	0.01	0.25	0.80
sTNFR1	-0.001 (-0.05 to 0.05)	-0.002	-0.03	0.98
**SCZ**^b^				
RANTES	-1.61 (-1.33 to 0.11)	-0.23	-2.25	0.03
IL-1RA	-0.11 (-0.50 to 0.28)	-0.17	-0.79	0.44
IL-18	0.02 (-0.02 to 0.06)	0.15	1.35	0.18
IL-18BP	0.003 (-0.02 to 0.02)	0.04	0.37	0.71
sTNFR1	-0.03 (-0.22 to 0.16)	-0.09	-0.39	0.70
**BD**^b^				
RANTES	-0.08 (-0.69 to 0.53)	-0.03	-0.36	0.72
IL-1RA	0.18 (-0.43 to 0.79)	0.19	0.84	0.41
IL-18	-0.003 (-0.03 to 0.03)	-0.02	-0.29	0.77
IL-18BP	-0.01 (-0.03 to 0.01)	-0.11	-1.28	0.20
sTNFR1	0.03 (-0.09 to 0.16)	0.16	0.72	0.48
**HC**^c^				
RANTES	-0.15 (-0.58 to 0.28)	-0.07	-0.90	0.37
IL-1RA	-0.05 (-0.31 to 0.21)	-0.06	-0.51	0.61
IL-18	0.002 (-0.02 to 0.02)	0.02	0.20	0.84
IL-18BP	0.003 (-0.02 to 0.03)	0.02	0.32	0.75
sTNFR1	-0.05 (-0.12 to 0.01)	-0.28	-2.11	0.04

*BD* Bipolar disorder, *CI* Confidence interval, *HC* Healthy participant group, *IL-18* Interleukin-18, *IL-18BP* Interleukin-18 binding protein, *IL-1RA* Interleukin-1 receptor antagonist, *RANTES* Regulated on activation normal T cell expressed and secreted, *SCZ* Schizophrenia or schizophreniform disorder, *sTNFR1* Soluble tumor necrosis factor receptor 1

^a^Separate regression for each immune marker, also controlled for sex, age, diagnosis, BMI, and smoking

^b^Separate regression for each immune marker, also controlled for sex, age, BMI, smoking, psychotropic medication, manic symptoms, and depressive symptoms

^c^Separate regression for each immune marker, also controlled for sex, age, BMI, and smoking

### Associations between impulsivity and psychopharmacological treatment

Among the patients, impulsivity was negatively associated with DDD of lithium (β = -0.19, t = -3.00, *p* = 0.003) and positively associated with DDD of antidepressants (β = 0.16, t = 2.58, *p* = 0.011) after controlling for sex, age, diagnosis, other psychotropic medications, manic symptoms, and depressive symptoms. There were no significant associations between impulsivity and DDD of antipsychotics (β = 0.03, t = 0.40, *p* = 0.69) or anticonvulsants (β = -0.11, t = -1.75, *p* = 0.08). The follow-up analysis in the SCZ group did not reveal any significant associations between psychopharmacotherapy and impulsivity, although directions of effects and effect sizes were similar to the results in the total patient sample (Table 4). In the BD group, impulsivity was negatively associated with DDD of lithium (β = -0.20, t = -2.54, *p* = 0.0122), while there were no other significant associations between psychopharmacotherapy and impulsivity. Effect directions of DDD of lithium and antidepressants were consistent across analyses in the total patient sample, SCZ, and BD.

**Table 4:** Linear regressions of associations between psychopharmacotherapy and impulsivity among patients with severe mental disorders

	**Coefficient estimate (98.75% CI)**	**Standardized coefficient β**	**t**	**p**
**Total patient sample**^a^				
Antipsychotics, DDD	-0.01 (-0.02 to 0.03)	0.03	0.40	0.69
Anticonvulsants, DDD	-0.04 (-0.10 to 0.02)	-0.11	-1.75	0.08
Lithium, DDD	-0.08 (-0.15 to -0.01)	-0.19	-3.00	0.003^*^
Antidepressants, DDD	0.01 (0.001 to 0.07)	0.16	2.58	0.011^*^
**SCZ**^a^				
Antipsychotics, DDD	0.02 (-0.02 to 0.05)	0.12	1.13	0.26
Anticonvulsants, DDD	-0.03 (-0.16 to 0.09)	-0.07	-0.69	0.50
Lithium, DDD	-0.12 (-0.35 to 0.10)	-0.14	-1.40	0.17
Antidepressants, DDD	0.03 (-0.02 to 0.09)	0.15	1.49	0.14
**BD**^a^				
Antipsychotics, DDD	-0.01 (-0.06 to 0.04)	-0.03	-0.35	0.73
Anticonvulsants, DDD	-0.04 (-0.12 to 0.03)	-0.12	-1.45	0.15
Lithium, DDD	-0.08 (-0.16 to -0.0003)	-0.20	-2.54	0.0122^*^
Antidepressants, DDD	0.03 (-0.01 to 0.07)	0.14	1.82	0.07

*BD* Bipolar disorder, *CI* Confidence interval, *DDD* Defined daily dose, *SCZ* Schizophrenia or schizophreniform disorder, *YMRS* Young Mania Rating Scale

^a^Also controlled for sex, age, diagnosis, manic symptoms, and depressive symptoms

^*^p < 0.0125

## Discussion

We investigated links between impulsivity and circulating immune markers within putative pathophysiological pathways, and we examined associations between impulsivity and psychopharmacotherapy in severe mental disorders. The main findings were (1) no significant associations between circulating levels of RANTES, TNF, or IL-1 family immune markers and impulsivity, (2) a negative association between impulsivity and lithium treatment, and a positive association between impulsivity and antidepressant treatment.

We found a negative association between lithium treatment and impulsivity, which is in line with reported impulsivity-reducing properties of lithium in rodent models [22, 36]. While the exact molecular mechanisms that may underlie lithium effects are not fully understood, inhibition of glycogen synthase kinase-3 as well as of inositol monophosphatase and subsequent interplay with cellular signaling and neurotransmission have been identified as the main candidates [54–57]. Impulsivity-reducing properties of lithium treatment have also been described among patients with bipolar disorder in the context of manic episodes [38] or comorbid pathological gambling [58]. Moreover, impaired impulse control has been indicated as one of the major factors in suicidality [59], and lithium has shown protective effects on suicide risk in mood disorders [60–62]. On the other hand, a link between clinical characteristics and the lithium prescription practice [63] may also underlie the observed association between impulsivity and lithium treatment. The follow-up analyses revealed consistent effect directions across the total patient sample, SCZ, and BD, although the association in the SCZ group did not reach statistical significance. Therefore, it remains unclear to which degree the current findings apply to patients with schizophrenia or schizophreniform disorder.

The patient sample revealed a positive association between antidepressant treatment and impulsivity. This association might reflect a causal effect of antidepressants on impulsivity or a more intensive antidepressant prescription practice in impulsive patient populations. Indeed, clinical characteristics have been suggested to affect the antidepressant prescription practice, with deflections from the standard first-line treatment of major depressive disorder in more severely ill patient populations at higher suicide risk [64]. A large register-based observational study has shown an increased risk of suicide attempt repetition in individuals prescribed antidepressants, which was not apparent after accounting for the baseline risk of suicide attempt repetition [65]. However, meta-analyses of randomized controlled trials of antidepressants have indicated an increase in suicidality among adolescents [66] and young adults [67], while no significant increase was detected across the adult population [67]. Interestingly, it has been proposed that impulsivity may be particularly related to suicide risk among younger adults [68]. Importantly, antidepressants typically target serotonergic signaling, but the effects beyond reduction of depressive symptoms [69] and exact mechanisms of action remain elusive and likely complex [70, 71]. Of note, the follow-up analyses in the separate diagnostic groups revealed consistent effect directions and sizes but no statistically significant associations, highlighting the value of a trans-diagnostic approach but, at the same time, the need for large homogeneous samples.

There were no significant associations between anticonvulsant or antipsychotic treatment and impulsivity. This is in line with impulsivity reductions in rodents exposed to lithium but not in those exposed to anticonvulsants such as valproate or carbamazepine [35, 36]. However, clinical studies have previously indicated an inverse relationship between impulsivity and treatment with valproate [38] as well as antipsychotics [37]. These inconsistencies might reflect differences in the conceptualization of impulsivity, distinct characteristics of the patient populations, or pharmacological heterogeneity within the medication groups [72].

We tested the hypothesized associations between immune signaling and impulsivity across a broad spectrum of impulsivity levels, including impulsivity variance among healthy participants. While the current study captured elevated impulsivity across BD and SCZ disorders, no significant associations to the immune marker levels independent of diagnostic status were detected. This result is in contrast to earlier findings of links between the plasma level of the chemokine RANTES and impulsivity in individuals with alcohol dependence [20] and changes in impulsivity in rodents [22]. The rodent model of impulsivity has also shown parallel reductions in plasma IL-1β and impulsivity [22], but we found no corresponding associations between systemic signaling within IL-1 family, as reflected by IL-1RA, and impulsivity in the present study. Moreover, circulating levels of sTNFR1 were not significantly associated with impulsivity, in contrast to previous findings of a positive association between circulating TNF mRNA levels and impulsivity among individuals with suicidal behavior [21]. These disparities may indicate a relationship specific to certain populations, characterized by high substance use, suicide risk, or other distinct clinical features. Since some key immune markers such as IL-1β or TNF often circulate at levels just above the detection limit of commercially available assays and have relatively short biological half-life, we assessed the activity of IL-1 and TNF systems by using robust markers that are known to reflect the activity of these systems (i.e., sTNFR1, IL-1RA, IL-18, and IL-18BP) [25, 26]. However, the observed discrepancy may also be due to disparate sources (e.g., leukocytes, activated vascular endothelium, or fibroblasts) and expression patterns of these immune markers.

One of the strengths of the present study is a large well-characterized sample, which facilitated well-powered analyses of impulsivity levels across diagnostic categories and enabled the focus on associations with psychopharmacological treatment. Moreover, with a hypothesis-driven approach, we investigated candidate immune markers that have emerged across the clinical and experimental research fields. The current study should, however, be interpreted in light of its limitations. The cross-sectional observational design prevents inferences about causal directions, and the effects of confounding factors cannot be ruled out. We only studied one single measure of impulsivity (i.e., the total score of the BIS-11), which may not fully reflect the multifaceted construct of impulsivity [7]. Given the focus on individual candidate immune markers, investigation of the complex interplay within components of the immune system was outside the scope of the current study. Further, participants in the patient group had their blood drawn earlier in the day than healthy participants, which might potentially impact the results due to circadian variations in immune marker levels. However, the analyses were adjusted for diagnostic group. Moreover, degradation of some analytes before freezing of the samples cannot be dismissed. Furthermore, we used the prescribed dose of psychotropic medication as a proxy of the exposure to the psychotropic agent and thus were not able to account for possible pharmacokinetic influences or treatment non-compliance. Finally, the reduced sample sizes in the follow-up analyses of separate diagnostic groups challenge interpretation of the follow-up results and call for future investigations in larger homogenous samples.

## Conclusions

We show elevated impulsivity across BD and SCZ disorders but no significant associations between impulsivity and circulating immune markers within TNF and IL-1 superfamilies or RANTES. Interestingly, we found a significant negative relationship between impulsivity and lithium and a positive association with antidepressant treatment. Future investigations in clinical settings are warranted to determine the causal mechanisms of the observed associations between lithium and antidepressants and impulsivity.

### Supplementary Information

**Additional file 1: Table S1.** Demographic and clinical characteristics of the IL-1RA and sTNFR1 subsample.

## Data Availability

The datasets generated and/or analyzed during the current study are not publicly available due to privacy and ethical restrictions but are available from the corresponding author on reasonable request.

## Abbreviations

BD: Bipolar disorder diagnosis
BIS-11: Barratt Impulsiveness Scale
CDSS: Calgary Depression Scale for Schizophrenia
CRP: C-reactive protein
DDD: Defined daily dose
DSM-IV: Diagnostic and Statistical Manual of Mental Disorders Fourth Edition
ELISA: Enzyme-linked immunosorbent assay
GAF-F: Global Assessment of Functioning Split Version
IL: Interleukin
IL-1RA: Interleukin-1 receptor antagonist
IL-18BP: IL-18 binding protein
PANSS: Positive and Negative Syndrome Scale
RANTES: Regulated on activation normal T cell expressed and secreted
SCID-I: Structured Clinical Interview for DSM-IV axis I disorders
SCZ: Schizophrenia or schizophreniform disorder diagnosis
sTNFR1: Soluble TNF receptor 1
TNF: Tumor necrosis factor
TOP: Thematically Organized Psychosis
YMRS: Young Mania Rating Scale

## References

[1] McCutcheon RA, Reis Marques T, Howes OD (2020). Schizophrenia-an overview. JAMA Psychiat.

[2] Vieta E, Berk M, Schulze TG, Carvalho AF, Suppes T, Calabrese JR (2018). Bipolar disorders. Nat Rev Dis Primers.

[3] Åsbø G, Ueland T, Haatveit B, Bjella T, Flaaten CB, Wold KF (2022). The time is ripe for a consensus definition of clinical recovery in first-episode psychosis: suggestions based on a 10-year follow-up study. Schizophr Bull.

[4] Stanford MS, Mathias CW, Dougherty DM, Lake SL, Anderson NE, Patton JH (2009). Fifty years of the Barratt impulsiveness scale: an update and review. Person Individ Differ.

[5] Nanda P, Tandon N, Mathew IT, Padmanabhan JL, Clementz BA, Pearlson GD (2016). Impulsivity across the psychosis spectrum: Correlates of cortical volume, suicidal history, and social and global function. Schizophr Res.

[6] Fortgang RG, Hultman CM, van Erp TG, Cannon TD (2016). Multidimensional assessment of impulsivity in schizophrenia, bipolar disorder, and major depressive disorder: testing for shared endophenotypes. Psychol Med.

[7] Moeller FG, Barratt ES, Dougherty DM, Schmitz JM, Swann AC (2001). Psychiatric aspects of impulsivity. Am J Psychiatry.

[8] Moulin V, Golay P, Palix J, Baumann PS, Gholamrezaee MM, Azzola A (2018). Impulsivity in early psychosis: A complex link with violent behaviour and a target for intervention. Eur Psychiatry.

[9] Etain B, Lajnef M, Loftus J, Henry C, Raust A, Gard S (2017). Association between childhood dimensions of attention deficit hyperactivity disorder and adulthood clinical severity of bipolar disorders. Aust N Z J Psychiatry.

[10] The Network and Pathway Analysis Subgroup of the Psychiatric Genomics Consortium (2015). Psychiatric genome-wide association study analyses implicate neuronal, immune and histone pathways. Nat Neurosci.

[11] Gandal MJ, Zhang P, Hadjimichael E, Walker RL, Chen C, Liu S (2018). Transcriptome-wide isoform-level dysregulation in ASD, schizophrenia, and bipolar disorder. Science.

[12] Goldsmith DR, Rapaport MH, Miller BJ (2016). A meta-analysis of blood cytokine network alterations in psychiatric patients: comparisons between schizophrenia, bipolar disorder and depression. Mol Psychiatry.

[13] Marsland AL, Prather AA, Petersen KL, Cohen S, Manuck SB (2008). Antagonistic characteristics are positively associated with inflammatory markers independently of trait negative emotionality. Brain Behav Immun.

[14] Hjell G, Szabo A, Mørch-Johnsen L, Holst R, Tesli N, Bell C (2022). Interleukin-18 signaling system links to agitation in severe mental disorders. Psychoneuroendocrinology.

[15] Coccaro EF, Irwin M, Arevalo JMG, Dizon T (2021). Cole S.

[16] Larsen JB, Stunes AK, Vaaler A, Reitan SK (2019). Cytokines in agitated and non-agitated patients admitted to an acute psychiatric department: A cross-sectional study. PLoS ONE.

[17] Melhem NM, Munroe S, Marsland A, Gray K, Brent D, Porta G (2017). Blunted HPA axis activity prior to suicide attempt and increased inflammation in attempters. Psychoneuroendocrinology.

[18] Isung J, Aeinehband S, Mobarrez F, Nordström P, Runeson B, Asberg M (2014). High interleukin-6 and impulsivity: determining the role of endophenotypes in attempted suicide. Transl Psychiatry.

[19] Coccaro EF, Lee R, Coussons-Read M (2014). Elevated plasma inflammatory markers in individuals with intermittent explosive disorder and correlation with aggression in humans. JAMA Psychiat.

[20] Manzardo AM, Poje AB, Penick EC, Butler MG (2016). Multiplex immunoassay of plasma cytokine levels in men with alcoholism and the relationship to psychiatric assessments. Int J Mol Sci.

[21] Chang HB, Munroe S, Gray K, Porta G, Douaihy A, Marsland A (2019). The role of substance use, smoking, and inflammation in risk for suicidal behavior. J Affect Disord.

[22] Adams WK, Levesque DL, Cocker PJ, Kaur S, Bodnar TS, Young AH (2020). Decreased motor impulsivity following chronic lithium treatment in male rats is associated with reduced levels of pro-inflammatory cytokines in the orbitofrontal cortex. Brain Behav Immun.

[23] Sims JE, Smith DE (2010). The IL-1 family: regulators of immunity. Nat Rev Immunol.

[24] Aggarwal BB (2003). Signalling pathways of the TNF superfamily: a double-edged sword. Nat Rev Immunol.

[25] Arend WP (2002). The balance between IL-1 and IL-1Ra in disease. Cytokine Growth Factor Rev.

[26] Diez-Ruiz A, Tilz GP, Zangerle R, Baier-Bitterlich G, Wachter H, Fuchs D (1995). Soluble receptors for tumour necrosis factor in clinical laboratory diagnosis. Eur J Haematol.

[27] Miller AH (2020). Beyond depression: the expanding role of inflammation in psychiatric disorders. World Psychiatry.

[28] Cyran A, Pawlak E, Piotrowski P, Bielawski T, Samochowiec J, Tyburski E (2023). The deficit subtype of schizophrenia is associated with a pro-inflammatory phenotype but not with altered levels of zonulin: Findings from a case-control study. Psychoneuroendocrinology.

[29] Frydecka D, Krzystek-Korpacka M, Lubeiro A, Stramecki F, Stańczykiewicz B, Beszłej JA (2018). Profiling inflammatory signatures of schizophrenia: A cross-sectional and meta-analysis study. Brain Behav Immun.

[30] Stuart MJ, Baune BT (2014). Chemokines and chemokine receptors in mood disorders, schizophrenia, and cognitive impairment: a systematic review of biomarker studies. Neurosci Biobehav Rev.

[31] Merino JJ, Muñetón-Gomez V, Muñetón-Gómez C, Pérez-Izquierdo M, Loscertales M, Toledano Gasca A (2020). Hippocampal CCR5/RANTES elevations in a rodent model of post-traumatic stress disorder: Maraviroc (a CCR5 Antagonist) increases corticosterone levels and enhances fear memory consolidation. Biomolecules.

[32] Paris G, Bighelli I, Deste G, Siafis S, Schneider-Thoma J, Zhu Y (2021). Short-acting intramuscular second-generation antipsychotic drugs for acutely agitated patients with schizophrenia spectrum disorders. A systematic review and network meta-analysis. Schizophr Res.

[33] Yatham LN, Kennedy SH, Parikh SV, Schaffer A, Bond DJ, Frey BN (2018). Canadian Network for Mood and Anxiety Treatments (CANMAT) and International Society for Bipolar Disorders (ISBD) 2018 guidelines for the management of patients with bipolar disorder. Bipolar Disord.

[34] Leucht S, Leucht C, Huhn M, Chaimani A, Mavridis D, Helfer B (2017). Sixty years of placebo-controlled antipsychotic drug trials in acute schizophrenia: systematic review, bayesian meta-analysis, and meta-regression of efficacy predictors. Am J Psychiatry.

[35] Ohmura Y, Tsutsui-Kimura I, Kumamoto H, Minami M, Izumi T, Yamaguchi T (2012). Lithium, but not valproic acid or carbamazepine, suppresses impulsive-like action in rats. Psychopharmacology.

[36] Halcomb ME, Gould TD, Grahame NJ (2013). Lithium, but not valproate, reduces impulsive choice in the delay-discounting task in mice. Neuropsychopharmacology.

[37] Reddy LF, Lee J, Davis MC, Altshuler L, Glahn DC, Miklowitz DJ (2014). Impulsivity and risk taking in bipolar disorder and schizophrenia. Neuropsychopharmacology.

[38] Swann AC, Bowden CL, Calabrese JR, Dilsaver SC, Morris DD (2002). Pattern of response to divalproex, lithium, or placebo in four naturalistic subtypes of mania. Neuropsychopharmacology.

[39] Dalley JW, Roiser JP (2012). Dopamine, serotonin and impulsivity. Neuroscience.

[40] Icick R, Melle I, Etain B, Høegh MC, Gard S, Aminoff SR (2022). Preventive medication patterns in bipolar disorder and their relationship with comorbid substance use disorders in a cross-national observational study. Front Psychiatry.

[41] Kjelby E, Gjestad R, Sinkeviciute I, Kroken RA, Løberg EM, Jørgensen HA (2018). Trajectories of depressive symptoms in the acute phase of psychosis: Implications for treatment. J Psychiatr Res.

[42] American Psychiatric Association (1994). Diagnostic and statistical manual of mental disorders : DSM-IV.

[43] Owen MJ, Sawa A, Mortensen PB (2016). Schizophrenia. Lancet.

[44] First MB, Spitzer R, Gibbon M, Williams JBW (1995). Structured Clinical Interview for DSM-IV Axis I Disorders, Patient Edition, Version 2.

[45] Kay SR, Fiszbein A, Opler LA (1987). The positive and negative syndrome scale (PANSS) for schizophrenia. Schizophr Bull.

[46] Young RC, Biggs JT, Ziegler VE, Meyer DA (1978). A rating scale for mania: reliability, validity and sensitivity. Br J Psychiatry.

[47] Addington D, Addington J, Schissel B (1990). A depression rating scale for schizophrenics. Schizophr Res.

[48] Pedersen G, Hagtvet KA, Karterud S (2007). Generalizability studies of the global assessment of functioning-split version. Compr Psychiatry.

[49] Spitzer RL, Williams JB, Kroenke K, Linzer M, deGruy FV, 3rd Hahn SR (1994). Utility of a new procedure for diagnosing mental disorders in primary care The PRIME-MD 1000 study. Jama..

[50] Patton JH, Stanford MS, Barratt ES (1995). Factor structure of the Barratt impulsiveness scale. J Clin Psychol.

[51] Sanchez-Roige S, Fontanillas P, Elson SL, Gray JC, de Wit H, MacKillop J (2019). Genome-wide association studies of impulsive personality traits (BIS-11 and UPPS-P) and drug experimentation in up to 22,861 adult research participants identify Loci in the CACNA1I and CADM2 genes. J Neurosci.

[52] Reise SP, Moore TM, Sabb FW, Brown AK, London ED (2013). The Barratt impulsiveness scale-11: reassessment of its structure in a community sample. Psychol Assess.

[53] Lindstrøm JC, Wyller NG, Halvorsen MM, Hartberg S, Lundqvist C (2017). Psychometric properties of a Norwegian adaption of the Barratt Impulsiveness Scale-11 in a sample of Parkinson patients, headache patients, and controls. Brain Behav.

[54] Beurel E, Jope RS (2014). Inflammation and lithium: clues to mechanisms contributing to suicide-linked traits. Transl Psychiatry.

[55] Haggarty SJ, Karmacharya R, Perlis RH (2021). Advances toward precision medicine for bipolar disorder: mechanisms & molecules. Mol Psychiatry.

[56] Barkus C, Ferland JN, Adams WK, Churchill GC, Cowen PJ, Bannerman DM (2018). The putative lithium-mimetic ebselen reduces impulsivity in rodent models. J Psychopharmacol.

[57] Sakrajda K, Szczepankiewicz A (2021). Inflammation-related changes in mood disorders and the immunomodulatory role of lithium. Int J Mol Sci.

[58] Hollander E, Pallanti S, Allen A, Sood E, Baldini RN (2005). Does sustained-release lithium reduce impulsive gambling and affective instability versus placebo in pathological gamblers with bipolar spectrum disorders?. Am J Psychiatry.

[59] Nock MK, Hwang I, Sampson N, Kessler RC, Angermeyer M, Beautrais A (2009). Cross-national analysis of the associations among mental disorders and suicidal behavior: findings from the WHO World mental health surveys. PLoS Med.

[60] Cipriani A, Hawton K, Stockton S, Geddes JR (2013). Lithium in the prevention of suicide in mood disorders: updated systematic review and meta-analysis. BMJ.

[61] Tondo L, Vázquez GH, Baldessarini RJ (2021). Prevention of suicidal behavior in bipolar disorder. Bipolar Disord.

[62] Fitzgerald C, Christensen RHB, Simons J, Andersen PK, Benros ME, Nordentoft M (2022). Effectiveness of medical treatment for bipolar disorder regarding suicide, self-harm and psychiatric hospital admission: between- and within-individual study on Danish national data. Br J Psychiatry..

[63] Ko A, Swampillai B, Timmins V, Scavone A, Collinger K, Goldstein BI (2014). Clinical characteristics associated with lithium use among adolescents with bipolar disorder. J Child Adolesc Psychopharmacol.

[64] Fugger G, Bartova L, Fabbri C, Fanelli G, Dold M, Swoboda MMM (2022). The sociodemographic and clinical profile of patients with major depressive disorder receiving SSRIs as first-line antidepressant treatment in European countries. Eur Arch Psychiatry Clin Neurosci.

[65] Jakobsen SG, Larsen CP, Stenager E, Christiansen E. Risk of repeated suicide attempt after redeeming prescriptions for antidepressants: a register-based study in Denmark. Psychol Med. 2022:1–8. 10.1017/S0033291722002719. 10.1017/S0033291722002719 36043363

[66] Sharma T, Guski LS, Freund N, Gøtzsche PC (2016). Suicidality and aggression during antidepressant treatment: systematic review and meta-analyses based on clinical study reports. BMJ.

[67] Stone M, Laughren T, Jones ML, Levenson M, Holland PC, Hughes A (2009). Risk of suicidality in clinical trials of antidepressants in adults: analysis of proprietary data submitted to US Food and Drug Administration. BMJ.

[68] Dumais A, Lesage AD, Alda M, Rouleau G, Dumont M, Chawky N (2005). Risk factors for suicide completion in major depression: a case-control study of impulsive and aggressive behaviors in men. Am J Psychiatry.

[69] Stone MB, Yaseen ZS, Miller BJ, Richardville K, Kalaria SN, Kirsch I (2022). Response to acute monotherapy for major depressive disorder in randomized, placebo controlled trials submitted to the US Food and Drug Administration: individual participant data analysis. BMJ.

[70] Coccaro EF, Fanning JR, Phan KL, Lee R (2015). Serotonin and impulsive aggression. CNS Spectr.

[71] Jones JA, Zuhlsdorff K, Dalley JW (2021). Neurochemical substrates linked to impulsive and compulsive phenotypes in addiction: A preclinical perspective. J Neurochem.

[72] Möller HJ, Schmitt A, Falkai P (2016). Neuroscience-based nomenclature (jNbN) to replace traditional terminology of psychotropic medications. Eur Arch Psychiatry Clin Neurosci.

