# Supplementary material for: Impulsivity across severe mental disorders: a cross-sectional study of immune markers and psychopharmacotherapy
Source: BMC Psychiatry. 2023 Sep 7;23:659. doi: 10.1186/s12888-023-05154-4 (PMC10483855; doi:10.1186/s12888-023-05154-4)
Supplement: Supplementary file 1 — Additional file 1: Table S1. Demographic and clinical characteristics of the IL-1RA and sTNFR1 subsample. [file 12888_2023_5154_MOESM1_ESM.pdf]

Table S1 Demographic and clinical characteristics of the IL-1RA and sTNFR1 subsample

	**SCZ**	**BD**	**HC**	$\mathbf{p}^{\text{SCZ versus HC}}$	$\mathbf{p}^{\text{BD versus HC}}$	$\mathbf{p}^{\text{SCZ versus BD}}$
Total N = 240	N = 55	N = 32	N = 174			
**N (%)**						
Male	26 (76)	15 (47)	104 (60)	0.07	0.17	0.01
Use of antipsychotics	31 (91)	16 (50)	NA	NA	NA	<0.001
Use of anticonvulsants	1 (3)	6 (19)	NA	NA	NA	0.04
Lithium use	2 (6)	8 (25)	NA	NA	NA	0.03
Use of antidepressants	6 (18)	9 (28)	NA	NA	NA	0.31
**Median (IQR)**						
Age	25 (10)	30 (18)	31 (13)	$p^{\text{Kruskal-Wallis test}} = 0.18$		
BIS-11 total score	67 (18)	67 (20)	58 (11)	<0.001	<0.001	0.95
PANSS total score	63 (15)	39 (11)	NA	NA	NA	<0.001
CDSS	4 (6)	3 (4)	NA	NA	NA	0.99
YMRS	2 (3)	2 (4)	NA	NA	NA	0.86
GAF-F	40 (13)	54 (19)	NA	NA	NA	<0.001

BD bipolar disorder; BIS-11, Barratt Impulsiveness scale; CDSS, Calgary Depression Scale for Schizophrenia; GAF-F, Global Assessment of Functioning; HC, healthy participant group; IQR, interquartile range; NA, not applicable; PANSS, Positive and Negative Syndrome Scale; SCZ, schizophrenia or schizophreniform disorder; YMRS, Young Mania Rating Scale.

p values based on chi-squared-, Wilcoxon rank-sum-, or Kruskal-Wallis tests with post hoc pair-wise comparisons.
